# StrainInfo—the central database for linked microbial strain identifiers

**DOI:** 10.1093/database/baaf059

**Published:** 2025-09-24

**Authors:** Artur Lissin, Isabel Schober, Julius F Witte, Helko Lüken, Adam Podstawka, Julia Koblitz, Boyke Bunk, Peter Dawyndt, Peter Vandamme, Paul de Vos, Jörg Overmann, Lorenz C Reimer

**Affiliations:** Leibniz Institute DSMZ-German Collection of Microorganisms and Cell Cultures, Inhoffenstraße 7B, 38124 Braunschweig, Germany; Leibniz Institute DSMZ-German Collection of Microorganisms and Cell Cultures, Inhoffenstraße 7B, 38124 Braunschweig, Germany; Leibniz Institute DSMZ-German Collection of Microorganisms and Cell Cultures, Inhoffenstraße 7B, 38124 Braunschweig, Germany; Leibniz Institute DSMZ-German Collection of Microorganisms and Cell Cultures, Inhoffenstraße 7B, 38124 Braunschweig, Germany; Leibniz Institute DSMZ-German Collection of Microorganisms and Cell Cultures, Inhoffenstraße 7B, 38124 Braunschweig, Germany; Leibniz Institute DSMZ-German Collection of Microorganisms and Cell Cultures, Inhoffenstraße 7B, 38124 Braunschweig, Germany; Leibniz Institute DSMZ-German Collection of Microorganisms and Cell Cultures, Inhoffenstraße 7B, 38124 Braunschweig, Germany; Department of Mathematics, Computer Science and Statistics, Faculty of Sciences, Ghent University, Krijgslaan 281 - S9, 9000 Ghent, Belgium; Laboratory of Microbiology, Department of Biochemistry and Microbiology, Faculty of Sciences, Ghent University, K. L. Ledeganckstraat 35, 9000 Ghent, Belgium; Laboratory of Microbiology, Department of Biochemistry and Microbiology, Faculty of Sciences, Ghent University, K. L. Ledeganckstraat 35, 9000 Ghent, Belgium; Leibniz Institute DSMZ-German Collection of Microorganisms and Cell Cultures, Inhoffenstraße 7B, 38124 Braunschweig, Germany; Institute of Microbiology, Braunschweig University of Technology, Spielmannstraße 7, 38106 Braunschweig, Germany; Leibniz Institute DSMZ-German Collection of Microorganisms and Cell Cultures, Inhoffenstraße 7B, 38124 Braunschweig, Germany

## Abstract

Throughout scientific literature and databases, microbial strains are often distinguished using either non-standardized designations or one of several available culture collection numbers. The fact that different sources use different strain identifiers to describe the same strain significantly impedes the findability, reusability, and integration of published information, and also affects the reproducibility of results. In order to ensure the traceability of microbial strains, the new StrainInfo database presented in this work was developed based on the defunct StrainInfo.net portal to re-establish and further develop this important service for the collection and matching of all existing identifiers of microbial strains. New data is collected, standardized, and integrated from culture collection catalogues, sequence databases as well as the scientific literature. A new interface provides easy access to the identifiers, their interrelations, associated information, as well as links to additional data. To improve and encourage the referencing and linking of microbial strain data, StrainInfo has introduced the Digital Object Identifier as a persistent identifier for strains.

**Database URL**: https://straininfo.dsmz.de/

## Introduction

**Figure 1.**
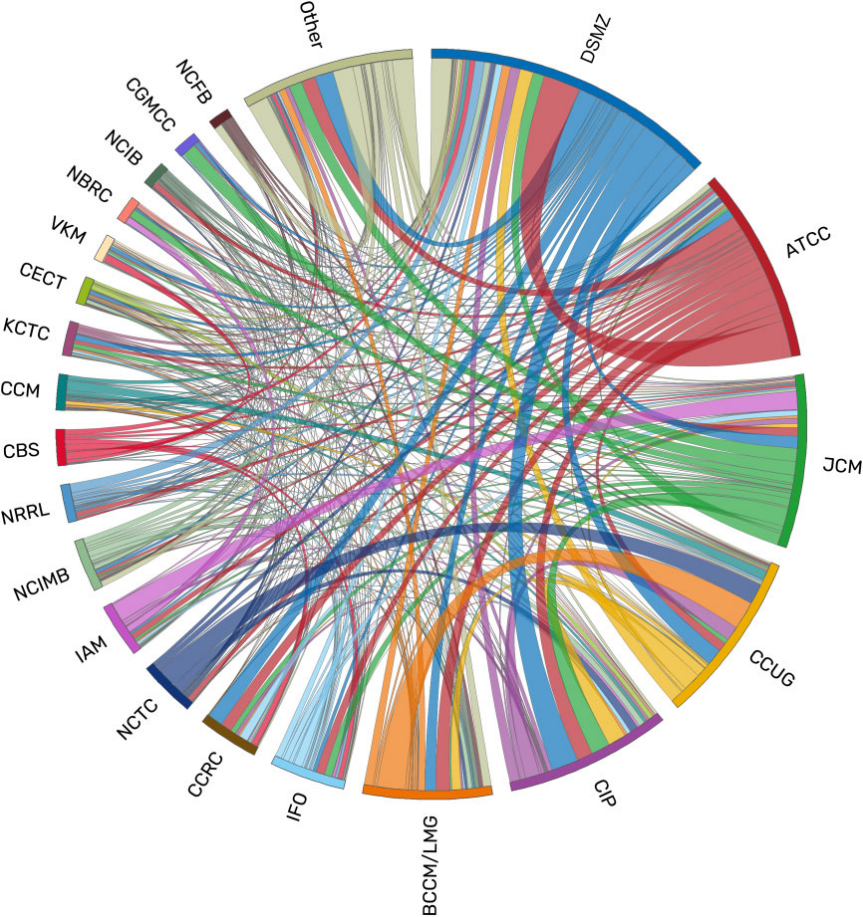
Exchange of cultures between culture collections according to StrainInfo data on deposition designations. Links are coloured by the providing collection. This image was constructed using Circos version v0.69–8 [28].

The findability of data and biological materials is essential for the reuse and the reproducibility of research results, but in microbiology it can be severely impeded if different designations and identifiers are used for the same strains. A microbial strain is defined as any culture that is derived from the same described and designated isolate, a pure culture derived from a single clone from a sample. Culture collections preserve and propagate strains and ensure the continuing availability of these highly valuable assets to the research community [1, 2]. In order to provide unambiguous identifiers, culture collection numbers are employed that are typically composed of a prefix specifying the collection and a catalogue-wide unique number (e.g. DSM 5522 or ATCC 43876). These culture collection numbers have the advantage of being unique and stable. Each culture of the same strain that is received by a collection is assigned a collection-specific number at deposition, resulting in several identifiers that are used interchangeably in literature and databases, as many strains are deposited in more than one collection. Strains of newly identified prokaryotic species or genera, for example, that are used for valid taxonomic descriptions are required to be deposited in at least two separate culture collections [3]. Culture collections also exchange cultures to ensure the broad availability of strains to the research community worldwide (Fig. 1). In addition, internal lab designations and even sequence accessions, for example for a 16S rRNA gene of the strain, are also commonly used to label strains. It is thus often difficult for researchers to link the differing identifiers and find all existing data belonging to the same strain.

The aim of the StrainInfo database is to resolve the links between the diverse culture collection numbers that exist for the same microbial strain, to provide a comprehensive resource for strain identifiers and thereby to facilitate the integration of strain-related information. It covers bacteria (39.7% of strains), archaea (0.3%), viruses (0.1%), as well as microbial fungi (48.9%) and algae (2.5%). StrainInfo defines a deposit as an instance of one strain that is maintained in one culture collection and has its own culture collection number (see also https://straininfo.dsmz.de/doc#manual_strains_deposits). The database links all microbial identifiers on the strain level by storing culture collection numbers, their relations, deposition-specific details, and associated information on taxonomy, sequence accessions, and literature.

The database builds on the idea and data of the defunct StrainInfo.net [4] website, which was developed at the BCCM/LMG Bacteria Collection of the Belgian Coordinated Collections of Microorganisms (BCCM/LMG, Ghent University) between 2000 and 2015. StrainInfo.net was a well-established resource and highly used in the microbiology community not only by individual users but also within workflows of tools and databases [5–8]. Due to lack of continued funding, development had to cease in 2015 and the database subsequently went offline. Within the recently established DSMZ Digital Diversity database platform (https://hub.dsmz.de), this important service could now be revived and further improved.

The new StrainInfo presented here uses data from the original StrainInfo.net database and combines it with newly collected data and newly programmed features. The information is provided through a modern and intuitive web interface, which enables users to easily find corresponding strain identifiers and links to associated data. A RESTful Application Programming Interface (API) allows for direct integration of strain identity resolution into workflows and other databases.

## Data

### StrainInfo.net data

The original StrainInfo.net data was extracted from the original Oracle database, newly structured and stored in a MariaDB 11 database consisting of currently 20 tables. The 796 965 strain identifiers in the database were sorted into culture collection numbers and other designations. To achieve this, all identifiers were assessed for possible prefixes, likely to be culture collection acronyms, and grouped according to these. The most abundant of the identified prefixes were manually reviewed and compared to The Global Registry of Scientific Collections (GRSciColl; https://scientific-collections.gbif.org) to connect them to culture collections and to identify the correct format of the respective culture collection numbers. This resulted in a list of 103 recognized culture collection number acronyms and formats that satisfy the following minimal requirements: (1) a unique acronym and (2) an identifier with at least three characters, at least one of which is numerical. This list and a tool for automatically identifying culture collection was published as ‘Collection Acronyms For Identification’ (*cafi*) [9]. Internally and for future strain-level matching, each culture collection number was saved as a tuple of four values: a unique culture collection ID (in place of the acronym), the identifier prefix, the identifier core number, and the identifier suffix. The prefix, which can for example denote subcollections, and the suffix, which can for example indicate variants, can be empty.

Verified culture collection numbers designate deposits. Other identifiers were counted as additional designations for the strain. Relationships between deposits from different collections were recreated based on the relation data from StrainInfo.net. Each strain in the database is labelled with a unique strain identifier (SI-ID). For the deposit level, a new identifier (SI-DP) was introduced. Deposition details were enriched by resolving given sequence accession numbers using NCBI E-utilities [10] and publication Digital Object Identifiers (DOIs) using the Crossref API (https://www.crossref.org). Taxonomic names were extracted and parsed using the Global Biodiversity Information Facility API Name Parser (https://www.gbif.org/tools/name-parser) and linked to the respective IDs of the List of Prokaryotic Names with Standing in Nomenclature (LPSN; [11]) and NCBI Taxonomy [12]. Culture collection numbers that could not be verified or that were ambiguously matched to strains, for example one deposit matching to more than one strain, were discarded. Finally, 430 260 deposits (previously known as ‘cultures’ in StrainInfo.net) of 272 341 strains formed the foundation for further developments.

### Gathering new data

To develop a sustainable update approach, we ask culture collections for permission to extract data from their web catalogues. Once permission is granted, this enables us to continuously integrate new data without the need for active participation by the collections.

For culture collections that offer an API to request data (such as the BioAware API used by BCCM/LMG or CIRM/CFBP), this is used to retrieve the data for all available deposits. As most of the collection catalogues do not provide programmatic access, however, the data is gathered by web scraping the catalogues. The HTML code of each catalogue web page is parsed and searched for strain identity information and deposition and deposit-specific details. The relevant data fields are extracted from the HTML code or API responses. Collection numbers are validated using the aforementioned *cafi* list of known culture collection number formats. Only data connected to a unique valid collection number is used for integration into the database. For each collection, the available data fields are investigated manually. Relevant data is subsequently extracted, validated using collection specific Python scripts and formatted into a standardized JSON structure. The following data was extracted, if available: the scientific name, type strain status, numbers in other culture collections and other designations of the deposit, sampling date, country, region, location and GPS coordinates, the institute of the isolator, the biological source material, the year of deposition in the particular collection, and the institute of the depositor. Encoded histories are also searched for these data points. The taxonomic names are verified using the same procedure as for existing StrainInfo.net data.

### Updating the database with new data

**Figure 2.**
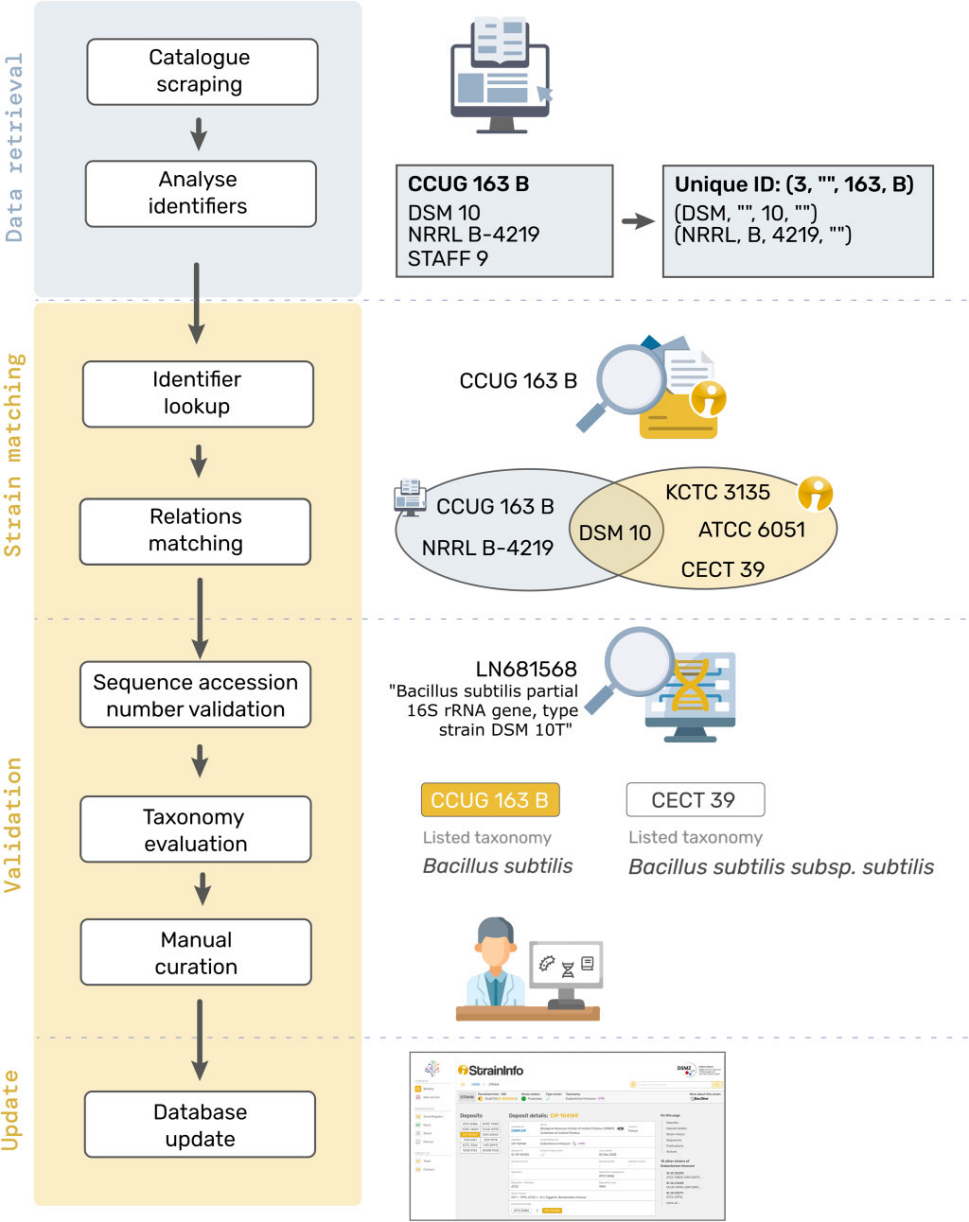
Schematic visualization of the process of matching new data to existing StrainInfo entries to update the database.

When new data gathered from culture collection catalogues is added to the database, a matching of the identifier needs to be performed to make sure that the data is correctly added either as an update to an existing deposit, as a new deposit of an existing strain, or as the first deposit of a new strain. The current approach of matching deposits on the strain level is still very similar to the one originally described for StrainInfo.net by Dawyndt *et al*. [13]. The culture collection number of the deposit is divided into a tuple containing the values for culture collection ID, identifier prefix, identifier core number, and identifier suffix (see Fig. 2, section ‘Data retrieval’). Any additional culture collection numbers listed for the strain are identified and verified using the *cafi* tool and stored as alternative designations in a candidate culture collection number tuple format.

In the ‘Strain matching’ step, the culture collection number tuple is searched against the tuples of all existing deposits in the database (see Fig. 2, section ‘Identifier lookup’). If a match is found, the corresponding deposit will be updated with the new information. Otherwise, the culture collection number tuple and the candidate culture collection tuples of other designations listed in the new deposit entry, are separately searched against all candidate culture collection number tuples of alternative designations stored in the database. If this ‘Relations matching’ (Fig. 2) produces a unique match, found in both or just one of the searches, a new deposit will be added to the respective existing strain. If no match can be found in these steps, a new strain with a first deposit will be created.

In a ‘Validation’ step (see Fig. 2), matches are further verified by comparing sequence accessions listed in the respective culture collection catalogue entries. Next the taxonomies listed are parsed as described for StrainInfo.net data, compared and evaluated. If any conflicts arise during these comparisons, the matching of a new deposit to an existing strain will be curated manually before integration of the new data.

**Figure 3.**
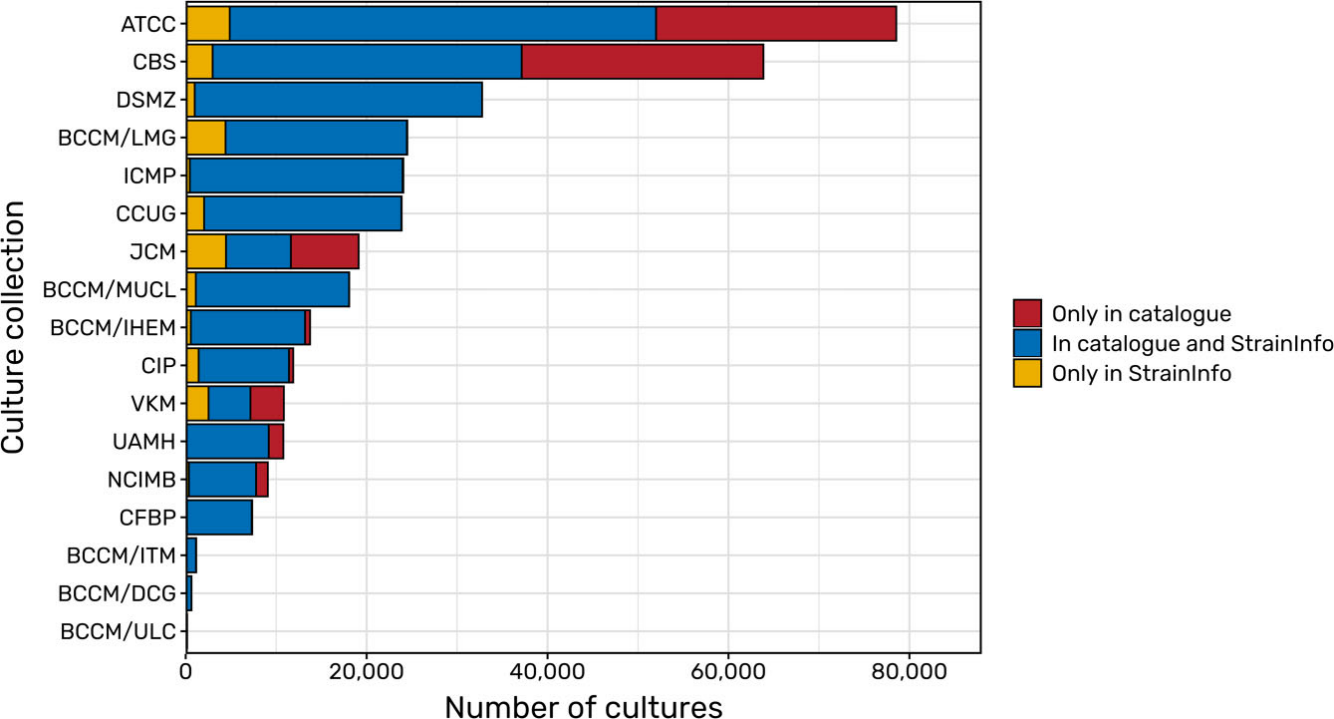
Comparisons of deposits of microbial strains (bacteria, archaea, viruses, as well as microbial fungi and algae) listed in the catalogues of selected culture collections and in StrainInfo. While blue areas represent the overlap of catalogues and the StrainInfo database, yellow areas show the amount of deposits that have been removed from public culture collection catalogues but can still be found in StrainInfo. Red areas show the number of deposits still missing in StrainInfo and thus the potential for future updates of the database.

Data from the DSMZ is added directly from internal databases. Additionally, the online catalogues of the BCCM collections, the Culture Collection of the University of Gothenburg (CCUG), and the International Collection of Microorganisms from Plants (ICMP) were scraped for data with the permission of the respective institutes. Further permissions have already been obtained, for example from the French Collection for Plant Associated Bacteria (CFBP), the CIRM-CF collection of filamentous fungi, and the Roscoff Culture Collection. Their catalogues contain a large amount of deposits that are not yet listed in StrainInfo (see Fig. 3). The upcoming integration of their data into the continuous update procedure of StrainInfo will ensure that data on new depositions and data changes will regularly be inserted and updated in the StrainInfo database.

### Sequence and literature data

Since the release of the first version of the new StrainInfo in September 2023, further cleaning steps and updates have been performed for the linked sequence data and publications. All 105 846 sequences in the StrainInfo database at the time and their links to strains were validated using the NCBI E-utilities [10], and the sequence description, sequence length or assembly level, and publication year was updated if necessary. Entries with NCBI GenBank [14] accessions that could not be resolved or are no longer listed as ‘live’ were removed from the StrainInfo database or were substituted with the given replacements. Nucleotide sequences that are part of a genome assembly were aggregated into assembly entries. Links of sequences to strains that could not be verified were dropped. The remaining and linked sequences were sorted into four groups: Complete genome sequences (‘Genome’, 633 assemblies), 16S rRNA gene, ITS, and other rRNA operon sequences (‘rRNA operon’, 18 551 sequences), gene sequences (‘Gene’, 38 553 sequences), and sequences related to patents (‘Patent’, 929 sequences). All sequences that did not fit in one of these categories, like genomic survey sequences, clone sequences, tRNA sequences, and others, were removed. In the future, the ‘Gene’ category might be further reduced to only include genes relevant for identification. In a first update, 25 202 complete genomes as well as 65 883 rRNA operon sequences were collected by going through current accession lists downloaded from the NCBI FTP server and programmatically matching them to StrainInfo strains by designation and species name, also considering all synonyms known in LPSN [11] and in the NCBI taxonomy [12].

Similarly, publications linked to strain designations were reviewed. As random manual checks showed that the former StrainInfo.net and also many collection catalogues link to publications on the species without reference to the specific strain, all literature data occurring in the original version of the database was discarded. To make new, strain-specific links to publications a custom script was employed that screens all species names in StrainInfo, searches PubMed [10] for publications mentioning the species name in the title and/or the abstract, downloads the respective metadata and abstracts and searches these for all culture collections numbers linked to the species name in the database.

### Current content

Currently, the database contains 717 337 strain identifiers of which 476 193 designate deposits with additional data. They match to 314 035 distinct strains, 20 065 of them type strains. 81.83% of strains have a taxonomy on the species level. Additionally, StrainInfo currently contains 117 118 sequence accessions and 36 846 publications.

### Introducing a new strain DOI

Due to the lack of a persistent identifier on the strain-level, strains are identified by deposit-level designations like culture collection numbers in literature and databases, irrespective of whether strains or specific deposits are being described. In StrainInfo we have introduced a new persistent identifier, describing the strain and encompassing all its deposits. To make sure that this identifier and all data related to it can always be cited in the future, it is registered for a DOI, which is composed of the StrainInfo-specific prefix, the SI-ID, and a version number (e.g. 10.60712/SI-ID34969.2). When changes are made to the strain data, the entry receives a new DOI with an updated version number. We propose the use of this strain DOI when referring to strains. The referencing of a universal strain identifier, by itself or besides a deposit identifier, in publications, databases, e.g. sequence databases, and culture collection catalogues, will considerably improve the findability of strains and of the data linked to them. The current and all previous versions of the strain data are saved in an archive from which they are downloadable as JSON-formatted files. This ensures that citations with DOIs can always be traced back to the status at the time of retrieval. StrainInfo DOIs are registered with rich metadata for optimal findability. These include subjects, creators and contributors with ORCID and ROR IDs and relations to other versions of the strain data.

## User interface

StrainInfo can be accessed through a user-friendly and well-structured web interface written in TypeScript using the Preact framework (Version 10).

**Figure 4.**
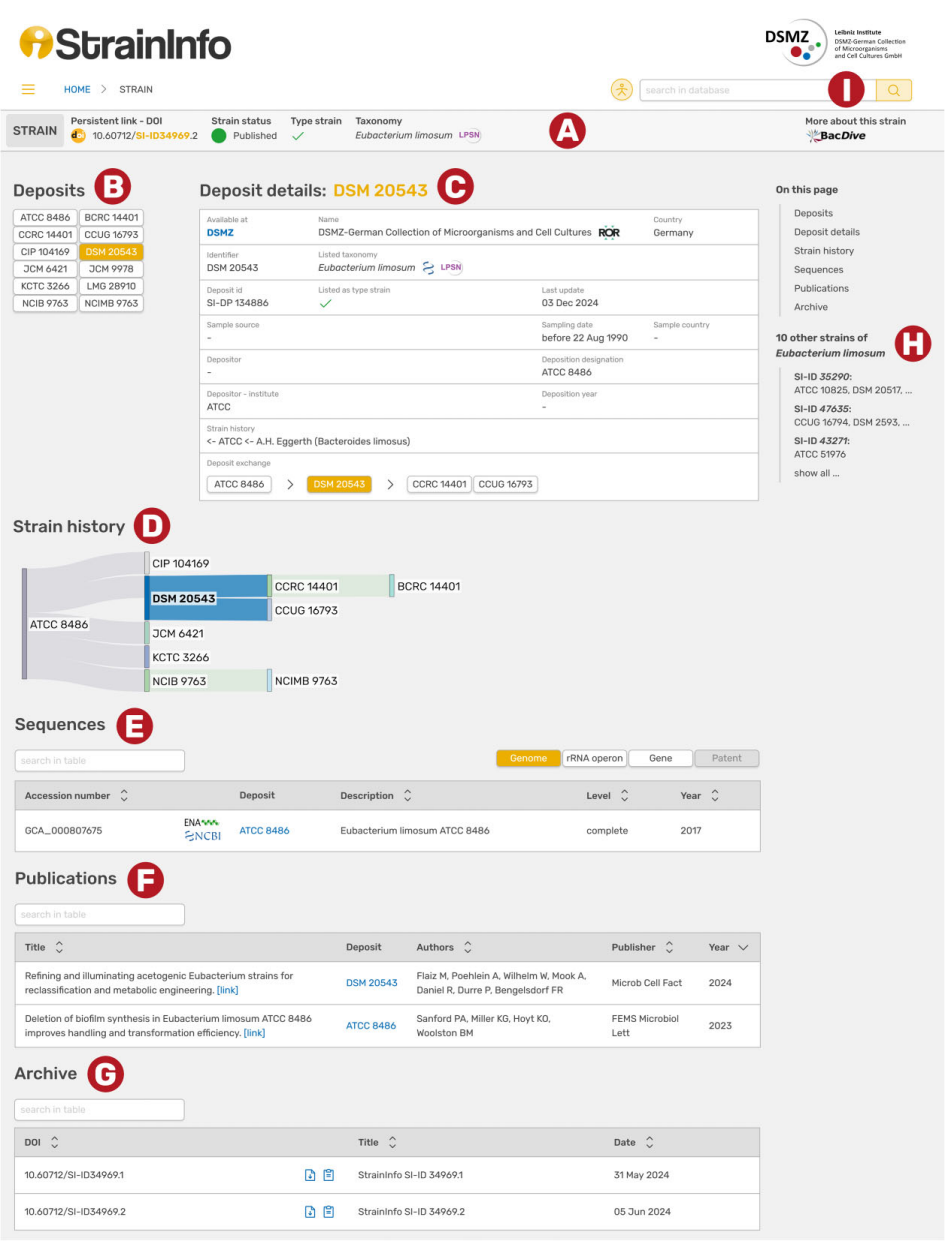
Screenshot of the StrainInfo strain page for strain SI-ID 34969 and its deposit DSM 20543. Each strain page consists of a header (A), a list of deposits belonging to the strain (B), deposition details of a selected deposit (C), the strain history (D), sequences of the strain (E), publications linked to the strain (F), and an archive (G). Navigation to other strains of the same species is possible through a list in the sidebar (H). Searches can be executed in the search bar on the top right (I).

The data is presented on individual strain pages consisting of a header and five different sections (Fig. 4). The header (A) contains the strain DOI with a copy-to-clipboard button, the deposition status and availability of the strain and information on taxonomy with a link to LPSN (if prokaryotic strains, otherwise NCBI Taxonomy is used) and the information whether the strain is a type strain. The catalogues of different culture collections do not always agree on the taxonomy of a strain. The taxonomy and type strain fields in the strain page header therefore show the most prevalent taxon name from the individual deposition details. On the far right of the header, a link to the Bac*Dive* [15] page of the strain is provided, where comprehensive isolation, physiological, and metabolic information can be found. Below the header is a list of all known designations for the strain (B). Deposits registered with deposition details in StrainInfo are shown as black text on white buttons. They can be clicked to open the respective deposition details. The deposit detail box (C) displays the information as it was listed in the respective culture collection catalogue at the time of data acquisition, which is the date given in the ‘Last update’ field. If accessible, the respective catalogue page is linked at the top of the box (‘Available at’). From the depositor and deposition designation information, the exchange history of the strain is deduced and displayed as a shallow history in the ‘Deposit exchange’ field. If available, the immediate ancestor and immediate descendants of the selected deposit are shown. The full reconstructed strain history is visualized below (D). The sequences section (E) lists all sequences linked to the strain in StrainInfo. They are shown in four groups: Complete genome sequences (‘Genome’), 16S rRNA gene, ITS, and other rRNA operon sequences (‘rRNA operon’), gene sequences (‘Gene’), and sequences related to patents (‘Patent’). For each sequence, links to the ENA [16] and NCBI GenBank [14] are provided. The publications section (F) lists literature linked to the strain and links to the publications via DOIs. All previous versions of the strain data can be found in the archive section (G) and downloaded in JSON format.

A list of other strains of the same species in the right sidebar (H) allows for easy navigation to closely related strains. Strains can generally be found through the search function at the top right (I). The search bars feature autocomplete suggestions and allow searches by designation (culture collection numbers and other designations), taxonomy (genus or species names), sequence accession number, culture collection, StrainInfo strain ID (SI-ID), and StrainInfo deposit ID (SI-DP).

Additionally, the entire database can be queried through the strains table search (https://straininfo.dsmz.de/search). The table initially shows all strains in the database and can easily be searched by applying combinable filters for taxonomy, type strain status, availability, and designation (also partly, e.g. a culture collection acronym).

A documentation is provided on the ‘Manual’ page, where new users can, among other information, find the starting point of a guided tour through the website and a video tutorial for first steps to using the StrainInfo web services in Python.

## Programmatic access

The StrainInfo data can programmatically be accessed through a freely available RESTful API with the base URL https://api.straininfo.dsmz.de/. Currently, 25 endpoints support retrieval of database statistics, strain and deposit search, and download of detailed deposition data in JSON format. This backend is written in PHP 8.2 using the Slim micro framework for HTTP routing and Redis for caching the results. A comprehensive documentation, generated using OpenAPI Specification v3.1.1 [17], can be found on the Web service page on the StrainInfo website. Additionally, the Manual page includes short written examples and video tutorials on how to request information from the StrainInfo API using Python. This programmatic access is not only a convenient way for retrieving data from the database, but is also necessary for the integration of strain resolution data from StrainInfo into other databases, tools and workflows.

Within the DSMZ Digital Diversity infrastructure, which encompasses several databases of high relevance for the life sciences, including Bac*Dive* [15], BRENDA [18], LPSN [11], and SILVA [19], the StrainInfo database provides a central and specific service for the resolution of microbial strain identifiers. Bac*Dive* is the largest database for prokaryotic strain-level information in the world. Its strain pages now display a StrainInfo widget showing all known culture collection numbers of the strain [15]. In future developments of the Bac*Dive* interface, it is planned that StrainInfo data will replace Bac*Dive*’s own collection of alternative strain identifiers. SILVA, the core database for ribosomal RNA (rRNA) genes, uses StrainInfo to connect rRNA gene sequence accessions with cultivation and type strain statuses. This is an essential step in the curation process and labelling of taxonomic groups in the widely used SILVA taxonomy [20, 21].

## Discussion and outlook

Unlike other databases that collect deposit-specific information, such as the Global Catalogue of Microorganisms [22] or the MIRRI strains catalogue [23], StrainInfo is matching and resolving strain relations, and depicts information on both the strain and the deposit level, making it easy for users to understand which identifiers represent the same strain. Other databases using strain-level data do not match deposits themselves, but largely depend on the strain resolutions collected by StrainInfo. Another approach is the StrainSelect [24] knowledge graph, which was constructed for bacterial strains, deposits and sequences using strain resolution data from StrainInfo.net and other databases, as well as sequence alignments. It is however only available as a complete download for use in programmatic applications and has, as of the time of writing, not been updated since its original publication in 2022.

Overall the StrainInfo database is closing a big gap by providing a reliable resource for microbial strain identifiers and their relations. By helping researchers to easily find and connect microbial strain data for their research, in single cases as well as on a large scale, and by archiving data on deposits no longer listed in culture collection catalogues (Fig. 3), this database provides an important service on the way to FAIR [25] and open microbial research. Within the DSMZ Digital Diversity infrastructure, StrainInfo is the essential central resource for the resolution of microbial strain identifiers. By introducing a new persistent identifier on strain level, we additionally provide a mechanism to easily identify and connect microbial data throughout literature and databases in the future.

We strive to continuously update the database with additional information from culture collection catalogues and links to related data. As gathering and matching data from different culture collections is labourious, we are working on a second and complementary service to also tackle the issue of assigning persistent strain identifiers already during the deposition process. For this purpose, we are currently developing a strain registration and deposition management service, provided through a web portal named StrainRegistry, which will allow microbiologists to directly register the strains they want to deposit with strain meta- and deposition data (https://straininfo.dsmz.de/strainregistry).

## Data Availability

The code for the StrainInfo database can be found at https://github.com/LeibnizDSMZ/StrainInfo [26]. The list of ‘Collection Acronyms For Identification’, (*cafi*) is available at https://github.com/LeibnizDSMZ/cafi [9]. The ‘Strain Identification and Authentication Methods’ (*saim*) have been published in the following GitHub repository: https://github.com/LeibnizDSMZ/saim/ [27].
